# Supplementary material for: Polyglutamylation of microtubules drives neuronal remodeling
Source: Nat Commun. 2025 Jun 25;16:5384. doi: 10.1038/s41467-025-60855-6 (PMC12198417; doi:10.1038/s41467-025-60855-6)
Supplement: Supplementary file 1 — Supplementary Information [file 41467_2025_60855_MOESM1_ESM.pdf]

Supplementary Figures

Polyglutamylation of microtubules drives neuronal remodeling

Antoneta Gavoci, Anxhela Zhiti, Michaela Rusková, Maria M. Magiera, Mengzhe Wang, Karin A. Ziegler, Torben J. Hausrat, Anselm I. Ugwuja, Shreyangi Chakraborty, Stefan Engelhardt, Matthias Kneussel, Martin Balastik, Carsten Janke, Thomas Misgeld, Monika S. Brill

Supplementary Figures

Supplementary Figure 1. Confirmation of motor neuron specificity of the RiboTag approach

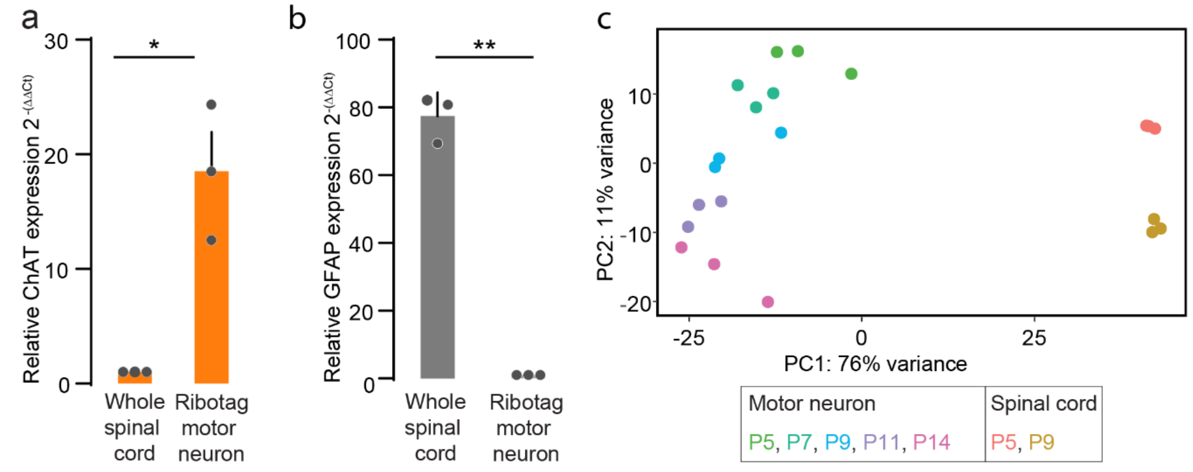

d

GO “Biological Process” in motor neuron vs spinal cord at P9

GO term	Description	Bonferroni
0000902	cell morphogenesis	6.25e-11
0006812	cation transport	2.03e-10
0035295	tube development	1.73e-9
0030198	extracellular matrix organization	3.97e-9
0048666	neuron development	4.03e-9
0031175	neuron projection development	4.13e-9
0061564	axon development	1.97e-8

e

GO “Cellular Component” in motor neuron vs spinal cord at P9

GO term	Description	Bonferroni
0045202	synapse	9.21e-6
0043209	myelin sheath	1.73e-5
0030424	axon	3.39e-4
0098793	presynapse	7.26e-4
0044297	cell body	2.12e-3
0015630	microtubule cytoskeleton	2.64e-3
0031252	distal axon	1.63e-2

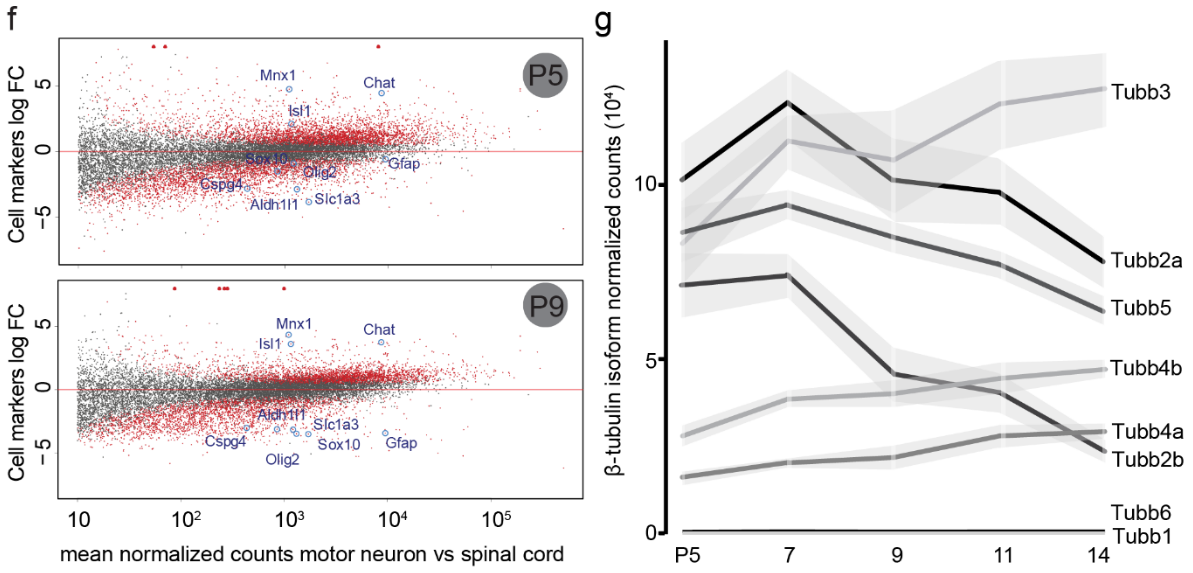

(a)-(b) RT-qPCR on whole spinal cord and motor neuron RiboTag ($Rpl22^{HA}$ + IP) samples of (a) choline acetyltransferase (*ChAT*; n = 3 RNA samples each) and **(b)** Glial glial fibrillary acidic protein (*GFAP*; n =

3 RNA samples each group). Results were calculated according to the 2-ΔΔCT method, using primers of a tested efficiency ≥ 90%. The amplification threshold cycle (CT) was the average of 2 or 3 technical replicates from each biological sample. **(c)** PCA plot of mRNA samples from the spinal cord and mRNA from IP from motor neurons. Individual dots represent a single animal, color-coded by age. **(d)** GO category "Biological Processes" generated using the top 500 most differentially expressed genes by comparing motor neurons against the spinal cord at P9 using a cutoff of $p_{adj} \leq$ 1e-7 and $|Log_2\ FC| > 2$. **(e)** GO category "Cellular Component", generated by using top 150 most differentially expressed genes by comparing motor neuron against spinal cord at P9 using a cutoff of $p_{adj} \leq$ 1e-7 and $|Log_2\ FC| > 2$. **(f)** MA plot at postnatal day (P) 5 and P9 highlights enrichment of motor neuron-specific cell markers in $Rpl22^{HA}$-positive motor neuron translatome samples, compared to the total spinal cord. Genes above the cutoff threshold of FDR ≤ 0.05 and a $|Log_2\ FC| \geq 1.5$ are depicted in red. Genes above the red horizontal line are enriched in the pull-down group, and genes below the red horizontal line are reduced. **(g)** The graph depicts normalized mRNA counts of beta-tubulin isotypes across the motor axon remodeling phase (postnatal day (P5, 7, 9, 11, 14). **Graphs:** mean + SEM, data representing single animals in **(a, b)** or mean + SEM in **(g)**. A two-tailed unpaired t-test determined significance: **, P < 0.01; ****, P < 0.0001. Source data are provided as a Source Data file.

Supplementary Figure 2. TTLL1^{mnWT} and TTLL1^{mnKO} normalization channels related to quantifications depicted in Figure 1

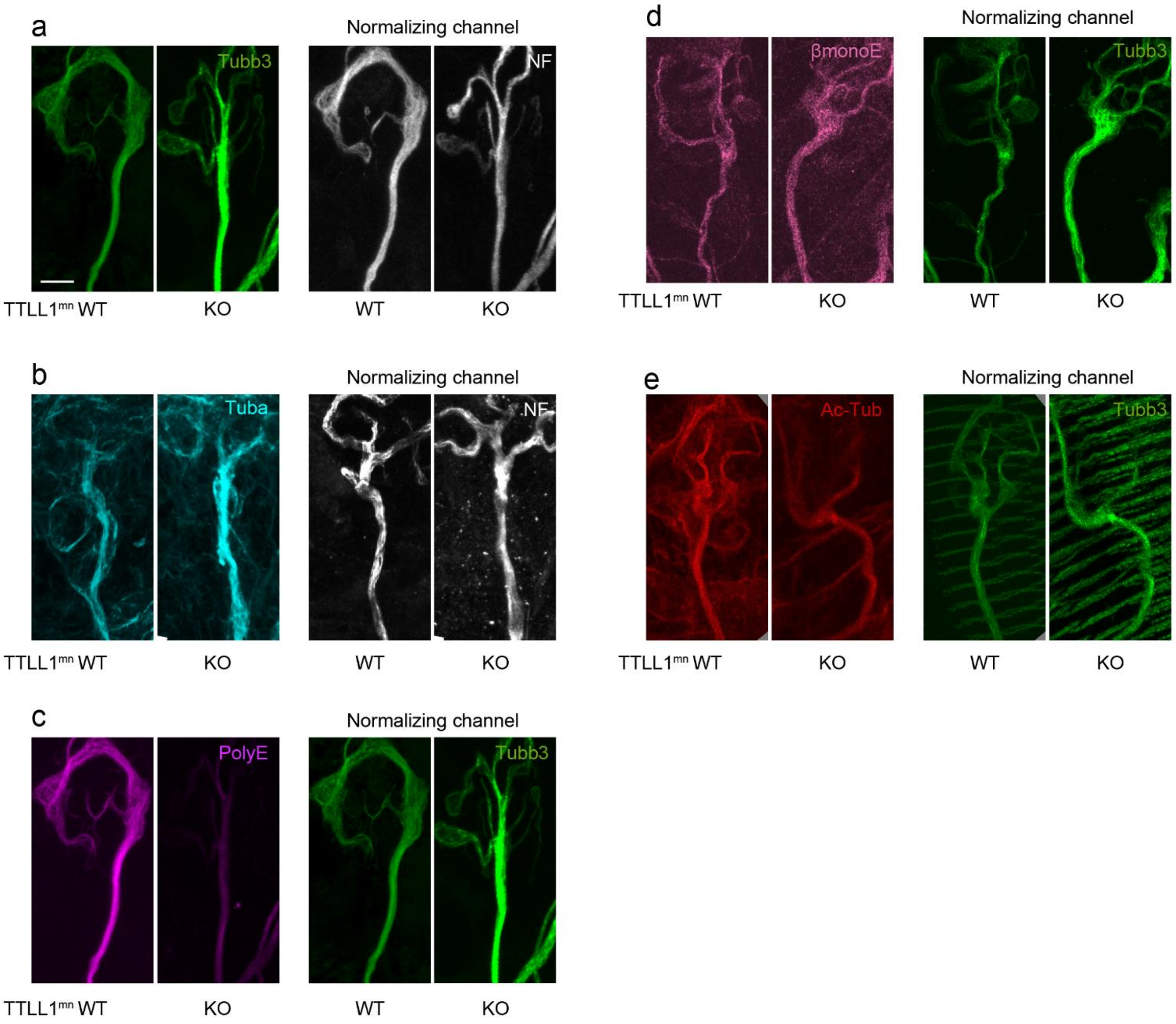

(a)-(e) Confocal stacks of NMJs with terminal axons depict immunostaining for microtubule markers (left; see Figure 2) and the normalizing channel (right) used for quantitative immunostainings on triangularis sterni muscles at P8-9 from in TTLL1^{mnWT} vs. TTLL1^{mnKO}. **(a)** Tubulin beta-3 staining (Tubb3, green) normalized on neurofilament (NF, white) staining (see Figure 1a and 1b). **(b)** Alpha-tubulin staining (Tuba, cyan) normalized on neurofilament (NF, white; see Figure 1c and 1d). **(c)** Polyglutamate chain staining (PolyE, magenta) normalized on tubulin beta-3 (Tubb3, green; Figure 1g and 1h). **(d)** Beta-tubulin isoform staining (βmonoE, pink) normalized on tubulin beta-3 (Tubb3, green; Figure 1i and 1j). **(e)** Acetylated tubulin staining (Ac-Tub, red) normalized on tubulin beta-3 (Tubb3, green; Figure 1k and 1l). Scale bar, 5 µm.

Supplementary Figure 3. Microtubule dynamics in terminal motor axons in explants of TTLL1^{mnKO}, TTLL7^{mnKO}, CCP1&6^{mnKO} crossbred to Thy1-EB3-YFP

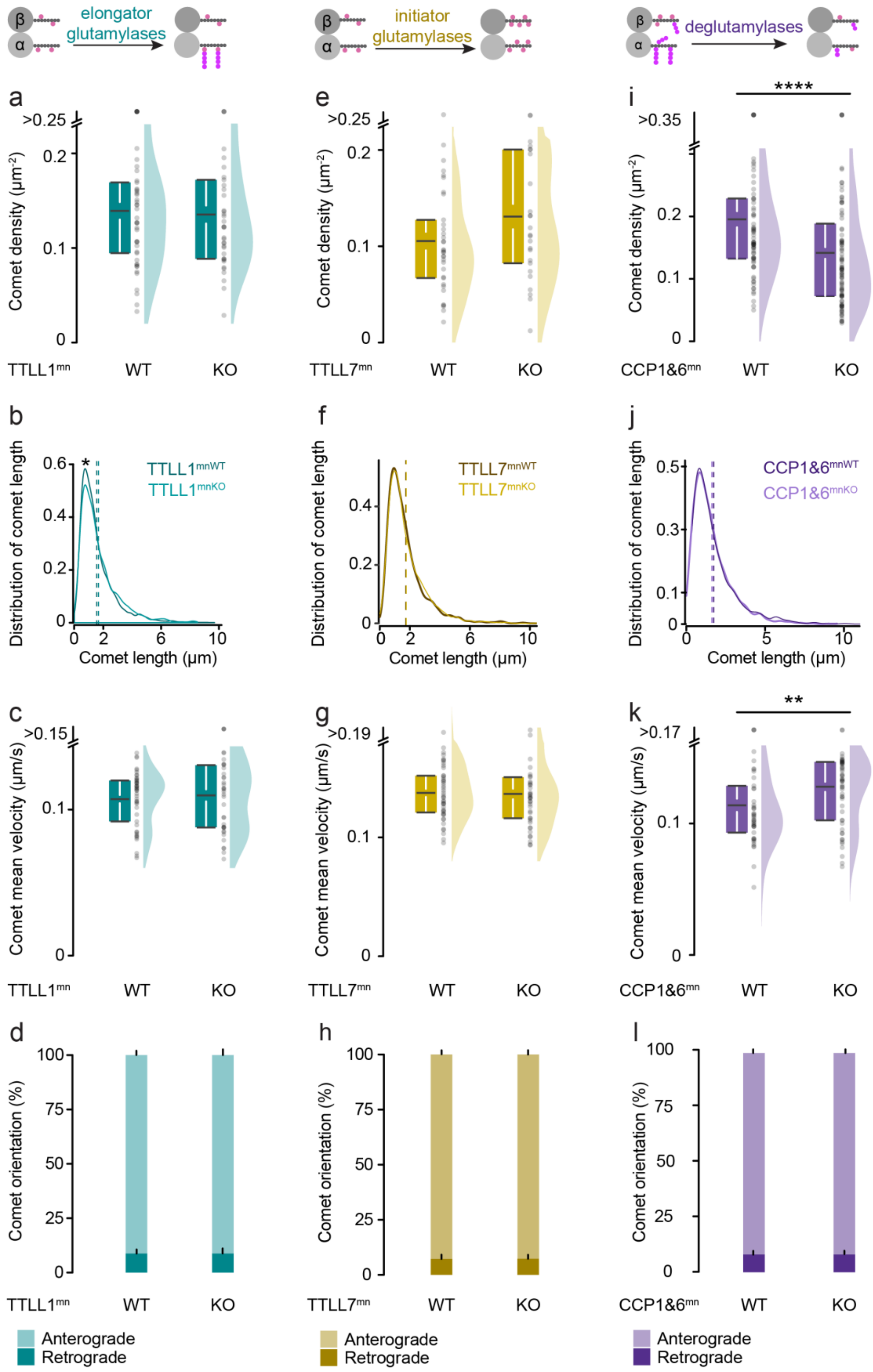

(a)-(l) Quantification of EB3 comet dynamics in P8-11 nerve-muscle explants derived from Thy1-EB3-YFP mice crossed to **(a)-(d)** TTLL1^{mnKO}, **(e)-(h)** TTLL7^{mnKO} and **(i-l)** CCP1&6^{mnKO} animals: **(a,e, i)** comet density, **(b,f,j)** distribution of comet lengths, **(c,g,k)** comet mean velocity and **(d,h,l)** orientation. ((a, c, d) WT n = 45 axons, 5 mice; KO n = 38 axons, 5 mice; (b) WT n =2287 comet tracks, 5 mice; KO n = 1752 comet tracks, 5 mice; p-value = 0.015; (e) WT n = 34 axons, 4 mice; KO n= 26 axons, 4 mice; (f) WT n = 2184 comet tracks, 4 mice; KO n = 2313 comet tracks, 4 mice; (g, h) WT n = 44 axons, 4 mice; KO n = 41 axons, 4 mice; (i) WT n = 74 axons, 12 mice; KO n = 96 axons, 14 mice, p-value = 5.45451E-5; (j) WT n = 1913 comet tracks, 10 mice; KO n = 1780 comet tracks, 10 mice; (k) WT n = 43 axons, 8 mice; KO n = 55 axons, 10 mice, p-value = 0.0075; (l) WT n = 55 axons, 11 mice; KO n = 68 axons, 11 mice. **Graphs:** 25% - 75% quantiles as a box with top and bottom black lines; mean as middle black line; SEM as white lines (left); data representing single axons as single dots (middle) and half violin (right) **(a,c,e,g,i,k)**, mean (dashed vertical line) and data (line) **(b,f,j)**, and mean + SEM **(d,h,l)**. A two-sided Mann-Whitney **(i, k)** and a Kolmogorov-Smirnov test **(b)** determined significance: *, P < 0.05; **, P < 0.01; ****, P < 0.0001. Source data are provided as a Source Data file.

Supplementary Figure 4. Genetic ablation of TTLL1, Tuba4a, or CCP1&6 does not cause a developmental phenotype in the neuromuscular system of postnatal mice

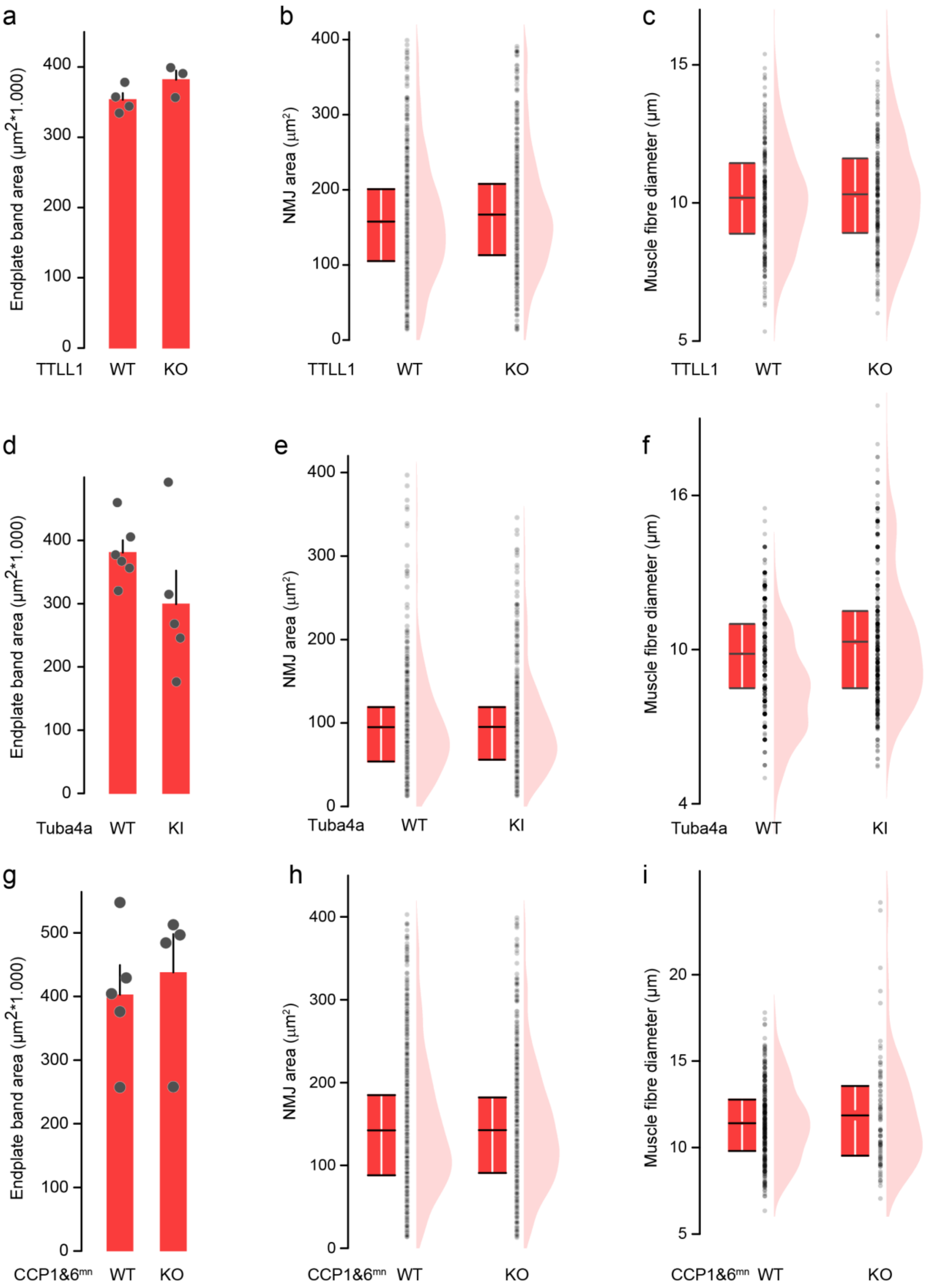

(a)-(i) Neuromuscular development assessed in triangularis sterni muscles at postnatal day (P) 6 from **(a) – (c)** $TTLL1^{KO}$ and $TTLL1^{WT}$ littermate controls, (**d**)-**(f)** $Tuba4a^{KI}$ and $Tuba4a^{WT}$ littermate controls, and **(g)-(i)** $CCP1\&6^{mnKO}$ and $CCP1\&6^{mnWT}$ littermate controls. **(a,d,g)** Endplate band area (a: WT n = 4 mice, KO n = 3 mice; d: WT n = 5 mice, KI n = 6 mice; g: WT n= 5 mice, KO n = 4 mice) and **(b,e,h)** NMJ area (b: WT n = 564 NMJs, 3 mice, KO n = 535 NMJs, 2 mice; e: n = 494 NMJs, 2 mice, KI n = 535 NMJs, 2 mice; h: n = 1116 NMJs, 5 mice, KO n = 820 NMJs, 4 mice) based on α-BTX staining. **(c,f,i)** Muscle fiber diameter based on phalloidin staining (**c,f**) or calnexin staining (**i**) (c: WT n = 235 muscle fibers, 3 mice, KO n = 203 muscle fibers, 4 mice; f: WT n = 447 muscle fibers, 4 mice, KO n = 399 muscle fibers, 3 mice; i: WT n = 291 muscle fibers, 5 mice, KO n = 87 muscle fibers, 4 mice). **Graphs:** mean + SEM, data points represent single animals **(a,d,g)** or 25% - 75% quantiles as box with top and bottom black lines; mean as middle black line; SEM as white lines (left); data representing single muscle fibers as dots (middle); half violin (right) **(b,c,e,f,h,i)**. A two-sided Mann-Whitney test determined no significant differences. Source data are provided as a Source Data file.

Supplementary Figure 5. TTLL7^{mnWT} and TTLL7^{mnKO} normalization channels related to quantifications depicted in Figure 3

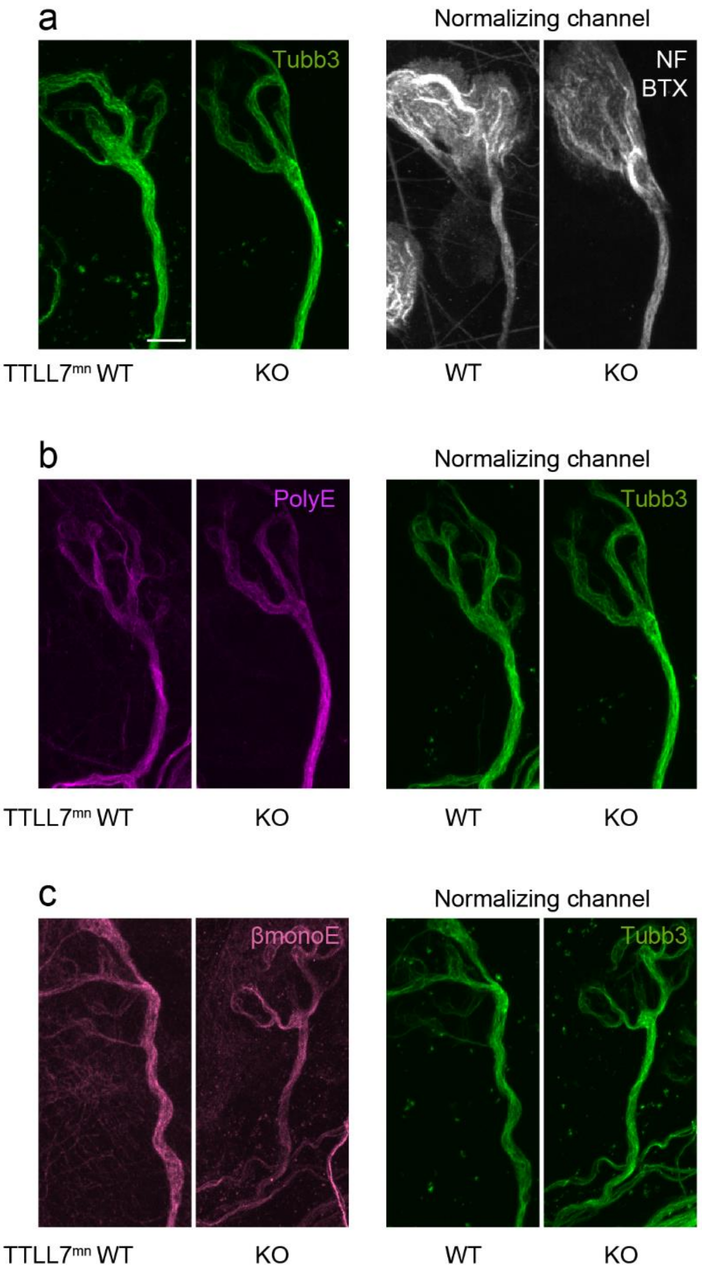

(a)-(c) Confocal stacks of NMJs with terminal axons depict immunostaining for microtubule markers (left; see Figure 3) and the normalizing channel (right) used for quantitative immunostainings on triangularis sterni muscles at P8-9 from in TTLL7^{mnWT} vs. TTLL7^{mnKO}. **(a)** Tubb3 (green) normalized on NF (white) staining (see Figure 3a and 3b). Note that α-BTX (white) labels the synapse only. **(b)** PolyE (magenta) normalized on Tubb3 (green; see Figure 3c and 3d). **(c)** βmonoE (pink) normalized on Tubb3 (green; see Figure 3e and 3f). Scale bar, 5 µm.

Supplementary Figure 6. TTLL1 and CCP1 CNS expression based on in-situ hybridization (adapted from Allen Brain Atlas)

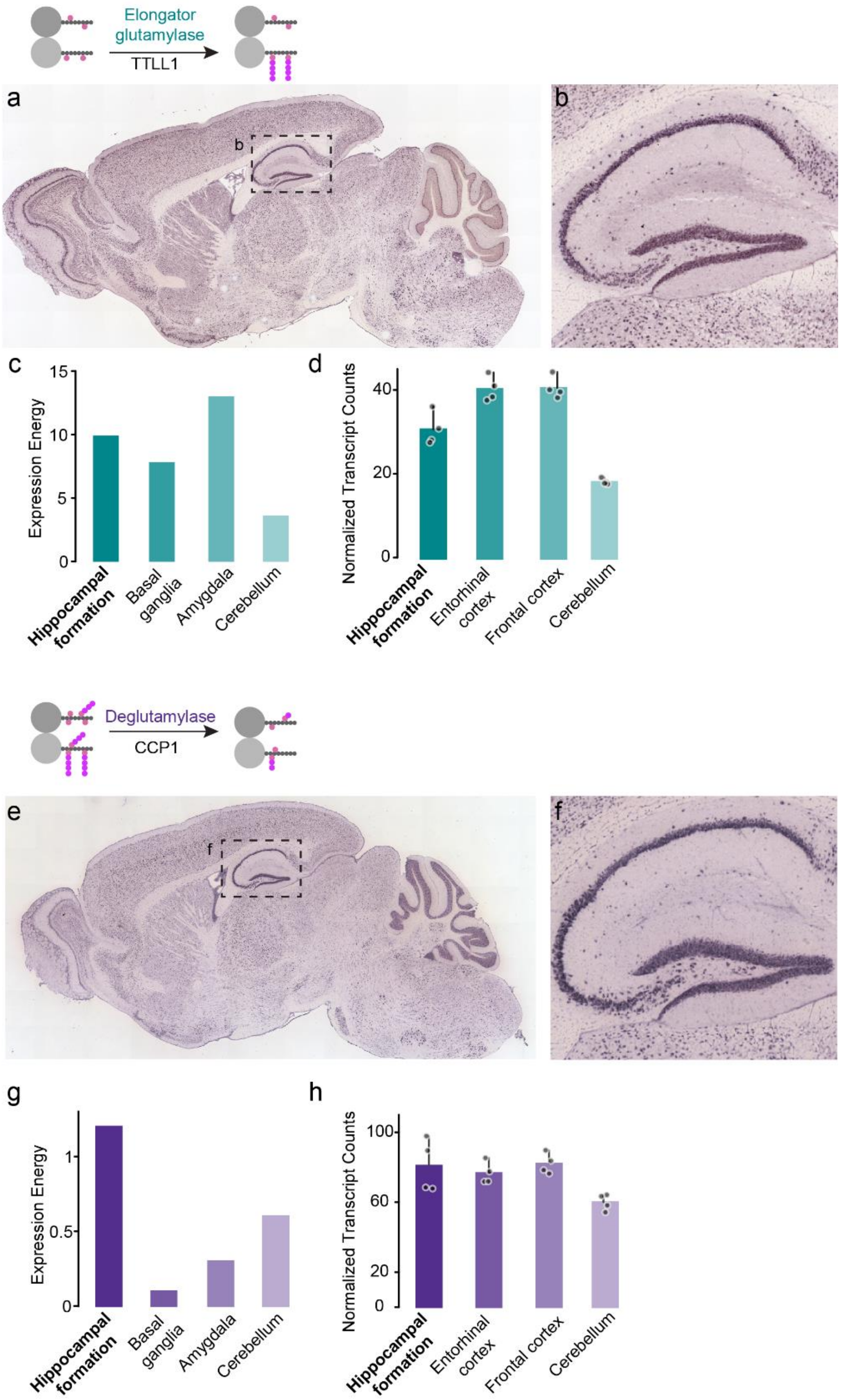

(a)-(h) CNS expression pattern based on in-situ hybridization for **(a)-(d)** TTLL1 and **(e)-(h)** CCP1. **(a,e)** Overview on 8 week-old murine sagittal brain sections for **(a)** TTLL1 and **(e)** CCP1 probes. **(b,f)** Higher magnification of boxed areas. **(c,d,g,h)** Expression energy **(c,g)** and normalized transcript counts **(d,h)** for **(c)-(d)** TTLL1 and **(g)-(h)** CCP1 reveal expression in hippocampal formation. Graphs: mean + SEM, data representing single animals **(d,h)**.

Supplementary Figure 7. Measurement of TTLL1^{KO} pruning phenotype - related to Figure 4

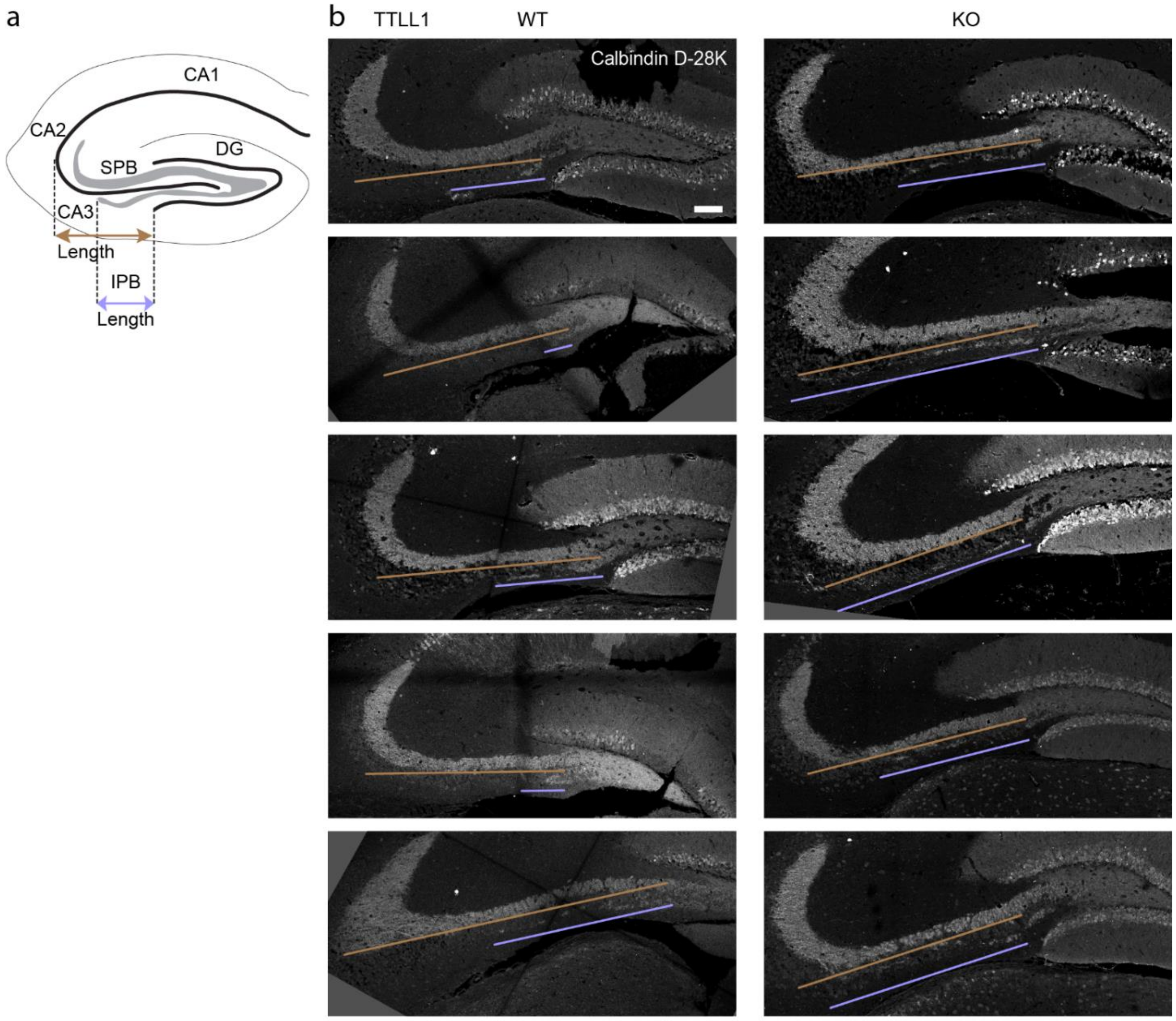

(a) Schematic of the hippocampus showing infrapyramidal bundle (IPB, gray), suprapyramidal main bundle (SPB, gray), dentate gyrus (DG), CA1, CA2, CA3 region; violet and brown lines indicate IPB and CA3 length measured **(b)** in 8 week-old TTLL1^{WT} (left) vs. TTLL1^{KO} littermate controls (right) based on Calbindin D-28K staining (white). Scale bar, 100 µm.

Supplementary Figure 8. Tuba4a^{WT} and Tuba4a^{KI} normalization channels related to quantifications depicted in Figure 5

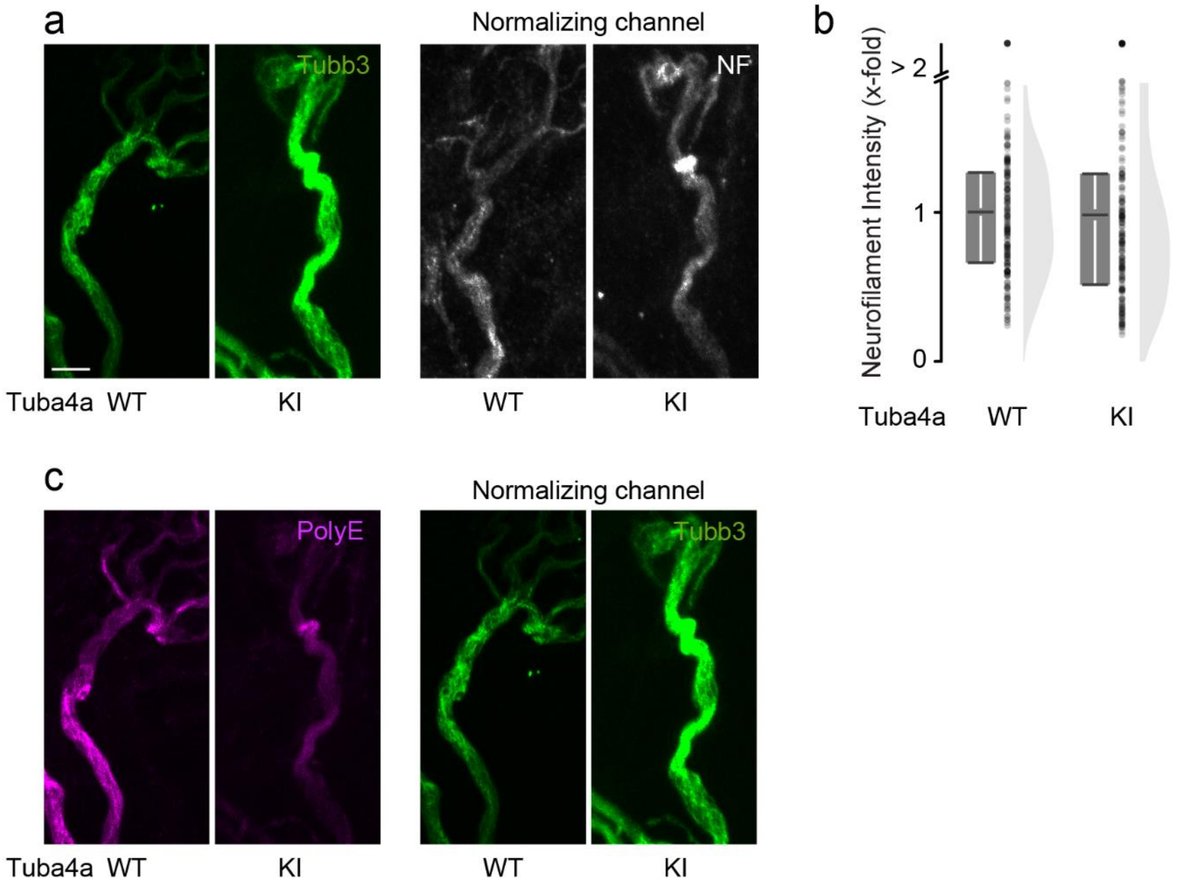

(a)-(c) Confocal stacks of NMJs with terminal axons depict immunostaining for microtubule markers (left; see Figure 5) and the normalizing channel (right) used for quantitative immunostainings on triangularis sterni muscles at postnatal day (P) 8-9 from in Tuba4a^{WT} vs. Tuba4a^{KI}. **(a)** Tubb3 (green) normalized on NF (white; see Figure 5b and 5c). **(b)** Quantification of NF intensity in terminal motor axons (WT n = 174 axons, 10 mice; KO n = 175 axons, 10 mice). **(c)** PolyE (magenta) normalized on Tubb3 (green; see Figure 5d and 5e). **Graph:** 25% - 75% quantiles as a box with top and bottom black lines; mean as middle black line; SEM as white lines (left); data representing single axons as single dots (middle); half violin (right). Scale bar, 5 µm. A two-sided Mann-Whitney test determined no significant differences. Source data are provided as a Source Data file.

Supplementary Figure 9. CCP1^{WT} vs. CCP1^{KO} and CCP1&6^{MNWT} vs. CCP1&6^{MNKO} normalization channels related to quantifications depicted in Figure 6

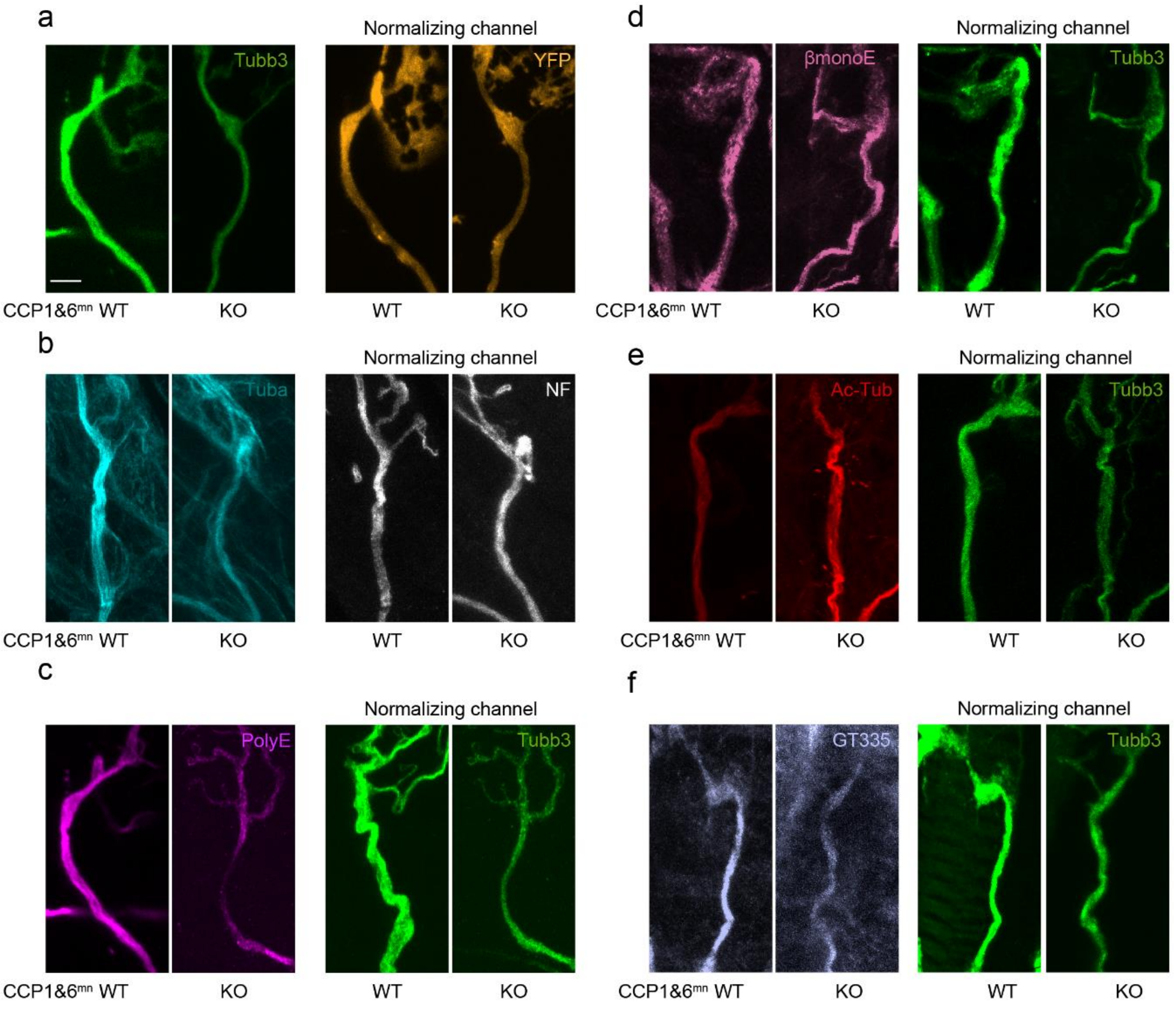

(a)-(f) Confocal stacks of NMJs with terminal axons depict immunostaining for microtubule markers (left; see Figure 6) and the normalizing channel (right) used for quantitative immunostainings on triangularis sterni muscles at postnatal day (P) 8-9 from in CCP1&6^{WT} vs. CCP1&6^{KO}. **(a)** Tubb3 (green) normalized on yellow fluorescent protein (YFP, Thy1-YFP-16, orange; see Figure 6a and 6b). **(b)** Tuba (cyan) normalized on NF (white; see Figure 6c and 6d). **(c)** PolyE (magenta) normalized on Tubb3 (green; see Figure 6g and 6h). **(d)** βmonoE (pink) normalized on Tubb3 (green; see Figure 6i and 6j). **(e)** Ac-Tub (red) normalized on Tubb3 (green; see Figure 6k and 6l). **(f)** Staining for GT335 (light blue) normalized on Tubb3 (green; see Figure 6n and 6o). Scale bar, 5 µm.

Supplementary Figure 10. Correlative analysis of EB3-YFP comet to tubulin beta-3 intensity in CCP1&6^{mnKO} crossbred to Thy1-EB3-YFP

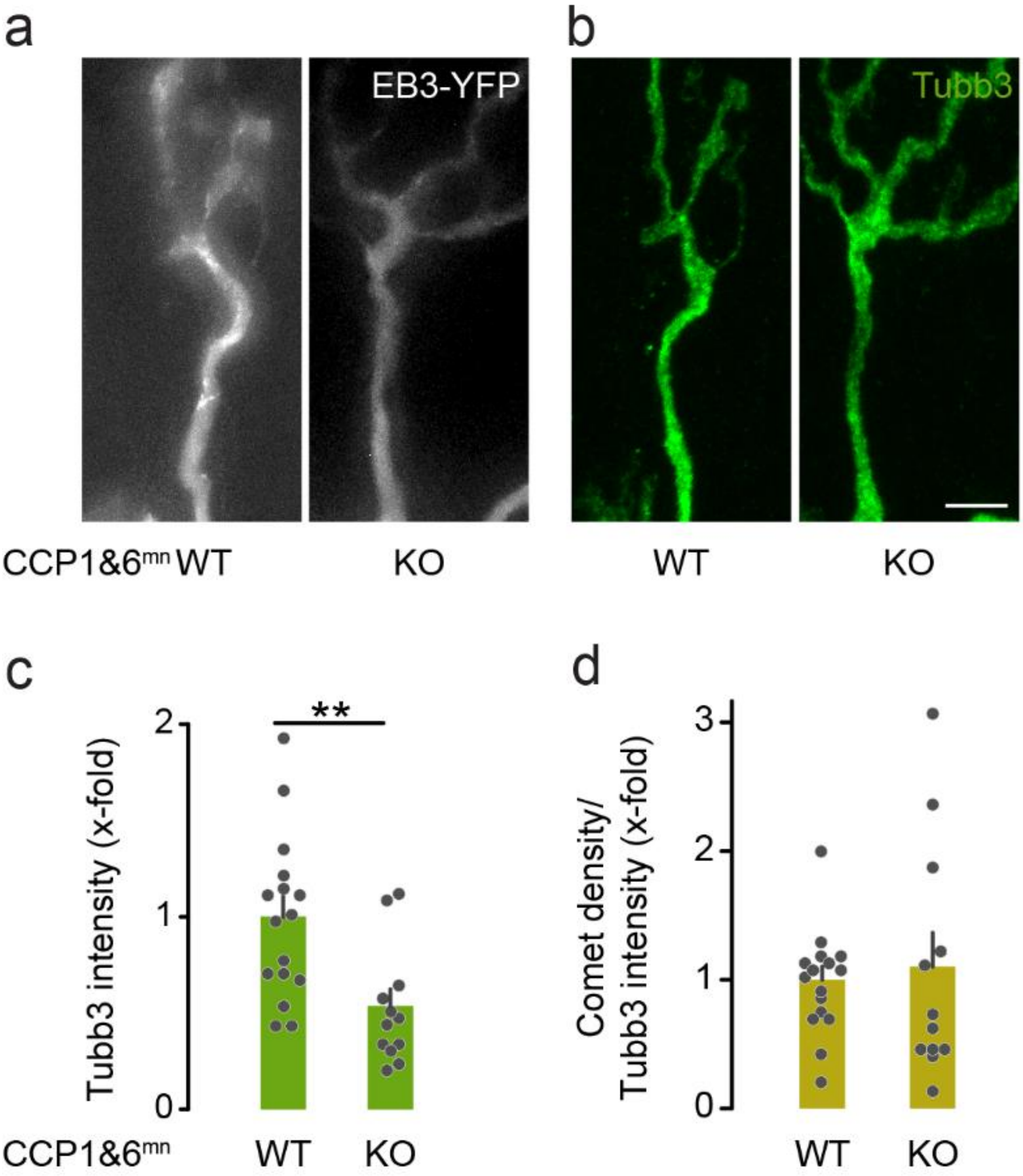

(a)-(b) Thy1-EB3-YFP motor axon live-imaging in nerve-muscle explants of postnatal day (P) 9-11 CCP1&6^{mnKO} and CCP1&6^{mnWT} littermates correlated to same axons fixed and immunostained. **(a)** Maximum intensity projection stacks of NMJs captured from time-lapse recording (20 s) of EB3-YFP comets (gray). **(b)** Confocal image of the same NMJ immunostained for Tubb3 (green). **(c)** Quantification of Tubb3 intensity (WT n = 16 axons, 3 mice, KO = 12 axons, 3 mice, p-value = 0.00129) and **(d)** ratiometric quantification of EB3 comet density to Tubb3 intensity (WT n = 16 axons, 3 mice, KO = 12 axons, 3 mice). **Graphs:** mean and SEM, data points represent single animals **(c,d)**. A two-sided Mann-Whitney test determined significance: **, $P < 0.01$. Scale bar, 5 µm. Source data are provided as a Source Data file.

Supplementary Figure 11. Measurement of CCP1^{KO} pruning phenotype - related to Figure 7

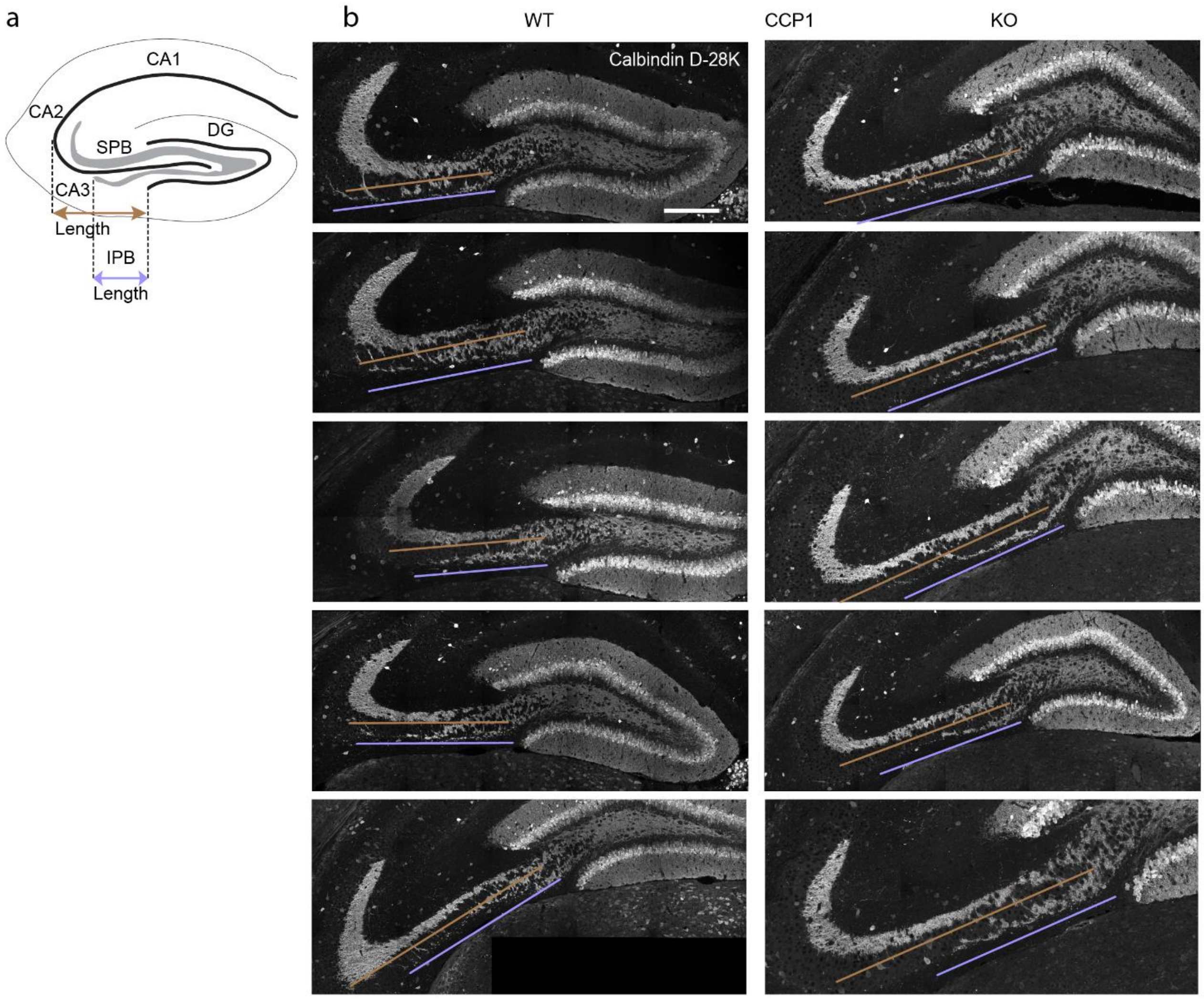

(a) Schematic of the hippocampus showing IPB (gray), SPB (gray), as well as DG and CA1, CA2, and CA3 regions; violet and brown lines indicate IPB and CA3 length measured **(b)** in P14 wildtype controls vs. CCP1^{KO} based on calbindin D-28K (white) staining. **(c)** Quantification of IPB length normalized to CA3 in P14 CCP1^{WT} vs. CCP1^{KO} littermates (n ≥ 6 hemispheres, 3 animals per genotype). **Graph:** mean + SEM, data points represent hemispheres. Scale bar, 100 µm.

Supplementary Figure 12. Severing enzymes in motor neuron translatome across neuronal remodeling and microtubule alterations in CCP1&6^{mnKO} adult motor neurons

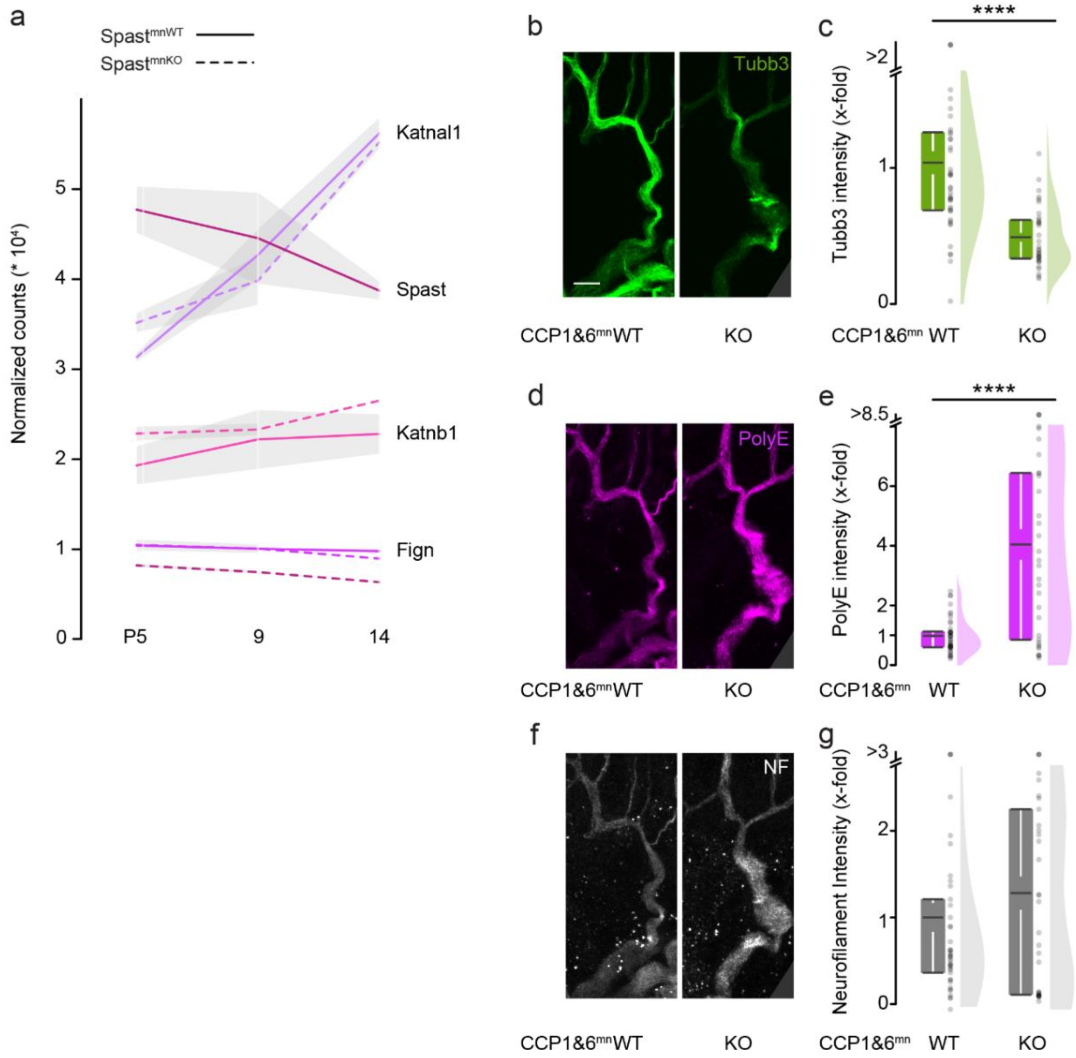

(a) Normalized read counts from translatome analysis of severing enzymes in motor neurons of Spast^{mnWT} or Spast^{mnKO} mice at postnatal day (P) 5, 9, and 14 (dashed line is Spast^{mnKO}, regular line Spast^{mnWT}). **(b)–(g)** Quantitative immunostainings for Tubb3, NF, and PolyE on triangularis sterni muscles at 6 weeks from CCP1&6^{mnKO} vs. CCP1&6^{mnKO} control littermates. **(b, d, f)** Confocal stack of a terminal axon leading to an NMJ depicting **(b)** Tubb3 (green), **(d)** PolyE (magenta), and **(f)** NF (white). **(c, e, g)** Quantification in terminal motor axons of **(c)** Tubb3, normalized to NF (WT: n = 42 axons, 3 mice, KO n = 33 axons, 3 mice, p-value = 5,65677E-8), **(e)** PolyE, normalized to Tubb3 (WT: n = 42 axons, 3 mice, KO n = 33 axons, 3 mice, p-value = 4,00404E-5) and **(g)** NF (WT: n = 42 axons, 3 mice, KO n = 33 axons, 3 mice). **Graphs:** 25% - 75% quantiles as a box with top and bottom black lines; mean as a middle black line; SEM as white lines (left); data representing single axons as single dots (middle); half violin (right) in **(c,e,g)**. A two-sided Mann-Whitney test determined significance: ****, P < 0.0001. Scale bar, 5 µm. Source data are provided as a Source Data file.

Supplementary Figure 13. BL6 and Spast^{KO} normalization channels related to quantifications depicted in Figure 9

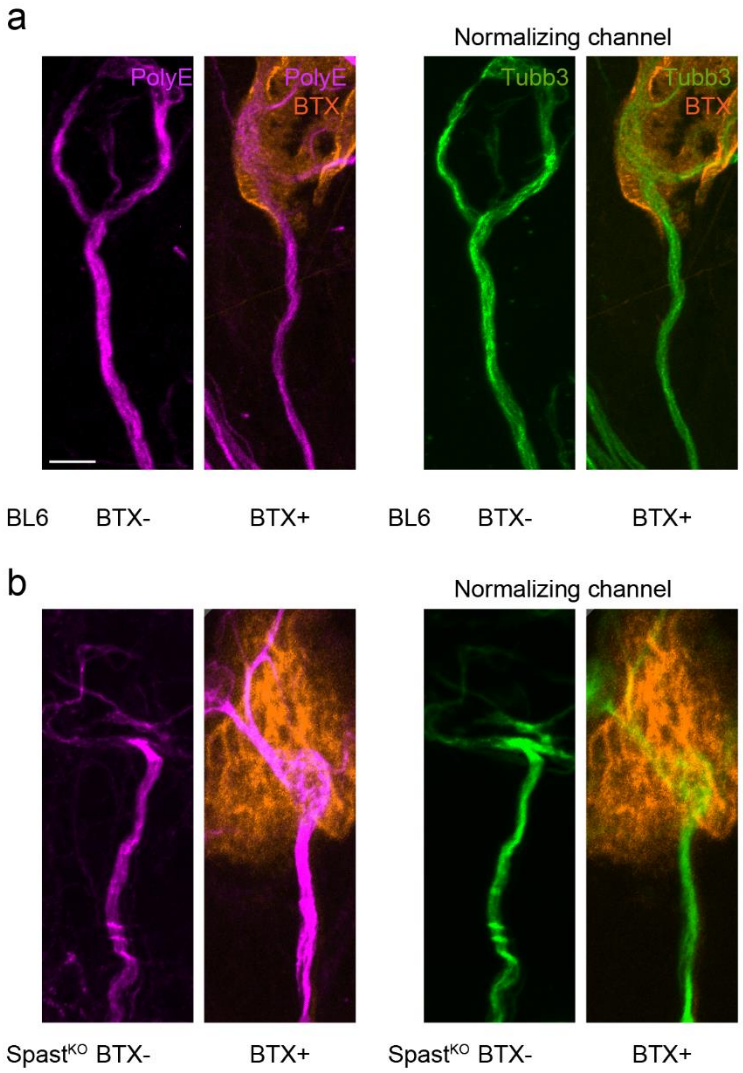

(a)-(b) Confocal stacks of NMJs with terminal axons depict immunostaining for microtubule markers (left; see Figure 8) and the normalizing channel (right) used for quantitative immunostainings on triangularis sterni muscles following α-BTX (orange) treatment. PolyE (magenta) normalized on Tubb3 (green) in **(a)** BL6 and **(b)** Spast^{KO} BTX-negative (BTX-) and BTX-injected (BTX+) terminal axons (see Figure 9c to 9f). Scale bar, 5 µm.
