# Supplementary material for: Polyglutamylation of microtubules drives neuronal remodeling
Source: Nat Commun. 2025 Jun 25;16:5384. doi: 10.1038/s41467-025-60855-6 (PMC12198417; doi:10.1038/s41467-025-60855-6)
Supplement: Supplementary file 2 — Description of Additional Supplementary Files [file 41467_2025_60855_MOESM2_ESM.pdf]

Description of Additional Supplementary Files

Supplementary Data 1: Gene differential expression between the whole spinal cord and RiboTag motor neuron samples

Supplementary Data 2: Motor neuron translatome during neuronal remodeling

Supplementary Data 3: Gene differential expression between $Spast^{mnWT}$ and $Spast^{mnKO}$

Supplementary Data 4: List of primers for genotyping.

Supplementary Movie 1: Microtubule dynamism in P9 Thy1-EB3-YFP x $CCP1\&6^{mnKO}$ motor neurons
