# Supplementary material for: Polyglutamylation of microtubules drives neuronal remodeling
Source: Nat Commun. 2025 Jun 25;16:5384. doi: 10.1038/s41467-025-60855-6 (PMC12198417; doi:10.1038/s41467-025-60855-6)
Supplement: Supplementary file 8 — Reporting Summary [file 41467_2025_60855_MOESM8_ESM.pdf]

nature portfolio

Corresponding author(s): Monika S. Brill

Last updated by author(s): Apr 26, 2025

Reporting Summary

Nature Portfolio wishes to improve the reproducibility of the work that we publish. This form provides structure for consistency and transparency in reporting. For further information on Nature Portfolio policies, see our Editorial Policies and the Editorial Policy Checklist.

Statistics

For all statistical analyses, confirm that the following items are present in the figure legend, table legend, main text, or Methods section.

n/a	Confirmed	
☐	☒	The exact sample size (*n*) for each experimental group/condition, given as a discrete number and unit of measurement
☐	☒	A statement on whether measurements were taken from distinct samples or whether the same sample was measured repeatedly
☐	☒	The statistical test(s) used AND whether they are one- or two-sided<br>*Only common tests should be described solely by name; describe more complex techniques in the Methods section.*
☒	☐	A description of all covariates tested
☐	☒	A description of any assumptions or corrections, such as tests of normality and adjustment for multiple comparisons
☐	☒	A full description of the statistical parameters including central tendency (e.g. means) or other basic estimates (e.g. regression coefficient) AND variation (e.g. standard deviation) or associated estimates of uncertainty (e.g. confidence intervals)
☐	☒	For null hypothesis testing, the test statistic (e.g. *F*, *t*, *r*) with confidence intervals, effect sizes, degrees of freedom and *P* value noted<br>*Give P values as exact values whenever suitable.*
☒	☐	For Bayesian analysis, information on the choice of priors and Markov chain Monte Carlo settings
☒	☐	For hierarchical and complex designs, identification of the appropriate level for tests and full reporting of outcomes
☒	☐	Estimates of effect sizes (e.g. Cohen's *d*, Pearson's *r*), indicating how they were calculated

Our web collection on statistics for biologists contains articles on many of the points above.

Software and code

Policy information about availability of computer code

Data collection	Confocal imaging: Leica Stellaris, Olympus FV3000 and Olympus FV1000 confocal.<br>Live imaging: epifluorescence microscope (Olympus BX51WI) equipped with, an automated filter wheel (Sutter Instruments; Lambda 10–3), a charge-coupled device camera (Visitron Systems; CoolSnap HQ2), controlled by µManager version 1.4.<br>qPCR: LightCyler 1.3 Real-Time PCR system (Roche S/N: 140 6143).<br>Motor neuron mRNA sequencing: Illumina HiSeq4000 Kit. RNA quantity and integrity was controlled with a Bioanalyzer (Agilent RNA 6000 Nano).<br>Injections: carried out with nanoliter injector (World Precision Instruments; Micro4 MicroSyringe Pump Controller connected with Nanoliter 2000) and guided by ultrasound (Visualsonics, Vevo® 2100).
Data analysis	Open source software: ImageJ/Fiji (version 1.52h), HTSeq-count and DESeq2 (version 1.24.0) and R Studio. Commercial software: GraphPad Prism (version 9.3.1) and Origin (2021b); Microsoft 365 apps Excel (version 2202); Adobe Photoshop CS5, (version 12.0); Adobe Illustrator CS5, (version 15.0.0). Neurolocida 360 v. 2017.01.4

For manuscripts utilizing custom algorithms or software that are central to the research but not yet described in published literature, software must be made available to editors and reviewers. We strongly encourage code deposition in a community repository (e.g. GitHub). See the Nature Portfolio guidelines for submitting code & software for further information.

Data

Policy information about availability of data

All manuscripts must include a data availability statement. This statement should provide the following information, where applicable:

- Accession codes, unique identifiers, or web links for publicly available datasets
- A description of any restrictions on data availability
- For clinical datasets or third party data, please ensure that the statement adheres to our policy

The immunostaining data generated in this study are provided in the Source Data file. The RNA-seq data generated in this study have been deposited in the Gene Expression Omnibus (GEO) database under accession number GSE296782 [https://www.ncbi.nlm.nih.gov/geo/query/acc.cgi?acc=GSE296782].

Research involving human participants, their data, or biological material

Policy information about studies with human participants or human data. See also policy information about sex, gender (identity/presentation), and sexual orientation and race, ethnicity and racism.

Reporting on sex and gender	n. a.
Reporting on race, ethnicity, or other socially relevant groupings	n. a.
Population characteristics	n. a.
Recruitment	n. a.
Ethics oversight	n. a.

Note that full information on the approval of the study protocol must also be provided in the manuscript.

Field-specific reporting

Please select the one below that is the best fit for your research. If you are not sure, read the appropriate sections before making your selection.

☒ Life sciences ☐ Behavioural & social sciences ☐ Ecological, evolutionary & environmental sciences

For a reference copy of the document with all sections, see nature.com/documents/nr-reporting-summary-flat.pdf

Life sciences study design

All studies must disclose on these points even when the disclosure is negative.

Sample size	No power calculation was performed to pre-determine sample sizes, but all sample sizes used in this study are comparable to those reported on similar previous studies (Wang, M. et al., J Cell Biol 2021, Brill, MS. et al., Neuron 2016). All numbers of axons and mice per genotype/ condition are reported on the figure legends/materials and methods.
Data exclusions	Low quality RNA (RIN < 8.5) was not sequenced. Lowly expressed RNAs (< 10 read counts) were excluded and differential gene expression analysis. Animals showing moderate to severe stress following BTX injection were excluded from further experimentation as described in the animal protocol.
Replication	Numbers of biological replicates used in this study are indicated in Figure legends. Three biological replicates per age group were tested in RNA sequencing experiments. All attempts at replication were successful.
Randomization	Experiments are performed only on littermate animals of specific age groups.
Blinding	Data collection and analysis was performed blind throughout the study.

Reporting for specific materials, systems and methods

We require information from authors about some types of materials, experimental systems and methods used in many studies. Here, indicate whether each material, system or method listed is relevant to your study. If you are not sure if a list item applies to your research, read the appropriate section before selecting a response.

Materials & experimental systems

n/a	Involved in the study
☐	☒ Antibodies
☒	☐ Eukaryotic cell lines
☒	☐ Palaeontology and archaeology
☐	☒ Animals and other organisms
☒	☐ Clinical data
☒	☐ Dual use research of concern
☒	☐ Plants

Methods

n/a	Involved in the study
☒	☐ ChIP-seq
☒	☐ Flow cytometry
☒	☐ MRI-based neuroimaging

Antibodies

Antibodies used

Primary antibodies:
1. anti-tubulin beta-3 (BioLegend #657404, 1:200, clone AA10),
2. anti-tubulin beta-3 (BD PharMingen #560339, 1:200, clone TUJ1),
3. anti-tubulin beta-3 (BioLegend #657405, 1:200, clone AA10),
4. anti-alpha-tubulin (Abcam #ab195889, 1:250, clone EP1332Y),
5. anti-Polyglutamylation Modification (Adipogen #AG-20B-0020, 1: 200, clone GT335),
6. anti-Polyglutamate chain (anti-polyE, Adipogen #AG-25B-0030, 1:1,000, polyclonal),
7. anti-alpha tubulin (acetyl K40, Abcam #ab24610, 1:1,000, clone 6-11B-1),
8. anti-neurofilament heavy polypetide (Abcam #ab4680, 1:500, polyclonal),
9. anti-βmonoE (Adipogen, AG-25B-0039-C050, 1:500),
10. anti-Choline Acetyl-Transferase (anti-ChAT, Novus Biologicals #NBP1-30052, 1:10, polyclonal),
11. anti-hemagglutinin (anti-HA, Sigma-Aldrich #H6908, 1:50, polyclonal),
12. anti-calbindin D-28K antibody (Swant #CB-38a, 1:300, polyclonal).

Secondary antibodies coupled to Alexa Fluor 488, Alexa Fluor 594, or Alexa Fluor 647:
anti-rabbit:
ThermoFisher #A-11070, 1:1,000, polyclonal;
ThermoFisher #A-11072, 1:1,000, polyclonal;
ThermoFisher #A-21246, 1:1,000, polyclonal;
ThermoFisher #A-32790, 1:1,000, polyclonal;
ThermoFisher #A-11037, 1:1,000, polyclonal;
ThermoFisher #A-11008, 1:1,000, polyclonal;
ThermoFisher #A-11037, 1:5000, polyclonal;
ThermoFisher #A-11008, 1:500, polyclonal.
anti-mouse:
ThermoFisher #A-11005, 1:1,000, polyclonal;
anti-chicken:
ThermoFisher #A-11042, 1:1,000, polyclonal;
ThermoFisher #A-21449, 1:1,000, polyclonal;
anti-goat:
ThermoFisher #A-11058, 1:1,000, polyclonal.

Validation

1. anti-tubulin beta-3 (BioLegend #657404, 1:200, clone AA10): Validation data provided by the supplier's Data sheet and cited in 4 publications (www.biolegend.com/de-de/products/alexa-fluor-488-anti-tubulin-beta-3-tubb3-antibody-9001);
2. anti-tubulin beta-3 conjugated to Alexa Fluor 555 (BD PharMingen #560339, mouse monoclonal, 1:200, clone TUJ1): Validation data provided by the supplier's Data sheet and cited in 3 publications (www.bdbiosciences.com/en-au/products/reagents/microscopy-imaging-reagents/immunofluorescence-reagents/alexa-fluor-555-mouse-anti-tubulin-class-iii.560339?tab=product_details);
3. anti-tubulin beta-3 conjugated tor Alexa Fluor 647 (BioLegend #657405, 1:200, clone AA10): Validation data provided by the supplier's Data sheet and cited in 4 publications (www.biolegend.com/en-gb/products/alexa-fluor-647-anti-tubulin-beta-3-tubb3-antibody-9848?GroupID=GROUP686)
4. anti-alpha-tubulin conjugated to Alexa Fluor 594 (Abcam #ab195889, 1:250, clone EP1332Y): Validation data provided by the supplier's Data sheet and cited in 11 publications (www.abcam.com/en-us/products/primary-antibodies/alexa-fluor-594-alpha-tubulin-antibody-dm1a-microtubule-marker-ab195889).
5. anti-Polyglutamylation Modification (Adipogen #AG-20B-0020, 1: 200, clone GT335): Validation data provided by the supplier and cited in 19 publications (https://adipogen.com/ag-20b-0020-anti-polyglutamylation-modification-mab-gt335.html).
6. anti-Polyglutamate chain (anti-polyE, Adipogen #AG-25B-0030, 1:1,000, polyclonal: Vaildation data provided by the supplier and cited in 19 publications (https://adipogen.com/ag-25b-0030-anti-polyglutamate-chain-polye-pab-in105.html).
7. anti-alpha tubulin (acetyl K40, Abcam #ab24610, 1:1,000, clone 6-11B-1): Validation data provided by the supplier and cited in 208 publications (www.abcam.com/products/primary-antibodies/alpha-tubulin-acetyl-k40-antibody-6-11b-1-ab24610.html#description_references).
8. anti-neurofilament heavy polypetide (Abcam #ab4680, 1:500, polyclonal): Validation data provided by the supplier and cited in 135 publications (www.abcam.com/en-us/products/primary-antibodies/neurofilament-heavy-polypeptide-antibody-ab4680).
9. anti-βmonoE (Adipogen, AG-25B-0039-C050, 1:500): Validation data provided by the supplier and cited in 1 publication (https://adipogen.com/ag-25b-0039-anti-beta-tubulin-beta-monoe-pab-in115.html).
10. anti-Choline Acetyl-Transferase (anti-ChAT, Novus Biologicals #NBP1-30052, 1:10, polyclonal): Validation data provided by the supplier and cited in 24 publications (www.novusbio.com/products/choline-acetyltransferase-chat-antibody_nbp1-30052#reviews-publications)

11. anti-hemagglutinin (anti-HA, Sigma-Aldrich #H6908, 1:50, polyclonal): Validation data provided by the supplier and cited in 1011 publications (www.sigmaaldrich.com/DE/en/product/sigma/h6908). Further validation was conducted on mouse tissue without HA anitgen.
12. anti-calbindin D-28K antibody (Swant #CB-38a, 1:300, polyclonal): Validation data provided by the supplier and cited in 44 publications (https://www.labome.com/product/SWant/CB38.html)

Animals and other research organisms

Policy information about studies involving animals; ARRIVE guidelines recommended for reporting animal research, and Sex and Gender in Research

Laboratory animals	The following mouse lines were used in this study at different ages (postnatal day 5, 7, 9, 11, 10, 13, 14 as well as 3 weeks, 5 weeks and 8 weeks). Experimental animals were kept together with littermates. The animals were housed in groups in individually ventilated cages (IVC) on dust-free wood chips with red plastic houses, paper rolls, gnawing sticks, wood wool, and fed with standard mouse chow. Ambient temperature in the cages was kept between 20 and 24 °C, humidity between 35 % and 55 %, and a light/dark cycle of 12hrs/12hrs. Mice were kept on a mixed genetic background and backcrossed to C57/Bl6N ~5-10 generations.<br>Motor neuron knockout-specific mice were generated by crossbreeding conditional knockout mice to ChAT-IRES-Cre mice, expressing Cre-recombinase under the ChAT promoter (Jackson #6410).<br>Ribotagging experiments were conducted on ChAT-IRES-Cre X homozygous Rpl22HA 36 mice (RiboTag flox/flox; Jackson, #11029) and conditional Spast flox/flox (generated in the laboratory of Matthias Kneussel, published in Brill et al., Neuron 2016).<br>Glutamylases (TTLL1 or TTLL7) or deglutamylases (CCP1 and CCP6) were deleted in motor neurons by crossbreeding conditional mutants of CCP1 & CCP6, TTLL1 or TTLL7 mice (from Janke lab, Institut Curie, Orsay) to ChAT-IRES-Cre animals. All animals were CCP1&6, TTLL1, or TTLL7 homozygous (flox/flox). Experimental animals – CCP1&6 motor neuron specific knockout (mnKO), TTLL1mnKO or TTLL7mnKO – were ChAT-IRES-Cre-positive, whereas littermate controls were ChAT-IRES-Cre-negative, named hereafter CCP1&6mnWT, TTLL1mnWT or TTLL7mnWT.<br>Thy1-YFP transgenic mice (cytoplasmic YFP in all motor neurons, Jackson #3709) were used to assess pruning speed in cross-breeding to CCP1&6mnKO, TTLL1mnKO, TTLL7mnKO, compared to their littermates CCP1&6mnWT, TTLL1mnWT, TTLL7mnWT.<br>Microtubule dynamics were visualized and analyzed by Thy1-EB3-YFP transgenic animals crossed to either CCP1&6mnKO, TTLL1mnKO, or TTLL7mnKO; CCP1&6mnWT, TTLL1mnWT, or TTLL7mnWT were littermate controls.<br>Conditional knockout of CCP1 and Spastin in motor neurons, monitored by ROSA-CAG-TdTomato reporter 74 (Ai14; Jackson; #7914; CCP1flox/flox X Spastflox/flox X TdTomato) was generated by injection of viral vectors encoding Cre-recombinase.<br>Block of neurotransmission was conducted and analyzed in constitutive spastin knockout (SpastKO) mice (homozygous Spast KO vs controls Spast WT) and C57BL/6N (Charles River, Strain Code 027) controls injected with α-BTX.<br>Spine density measurements and hippocampal pruning analysis were performed on TTLL1 constitutive knock-out at 3 and 8 weeks of age (3 and 8 weeks; named here TTLL1 KO; from Janke lab, Institut Curie, Orsay, France) backcrossed to CD-1 (Charles River, Strain Code 022) for three generations. TTLL1 KO and TTLL1 WT littermates were generated by crossbreeding conditional TTLL1 to Pgk1-cre 75 (Jackson, #020811; TTLL1KO/WT X TTLL1KO/WT).<br>CCP1 hippocampal pruning analysis was performed on constitutive knock-out (named here CCP1 KO; from Janke lab, Institut Curie, Orsay, France) at P14 generated by crossbreeding conditional CCP1 to CMV-cre expressing mice vs. P14 wildtype controls.<br>Cytoskeletal analysis and pruning speed were analyzed in homozygous tubulin alpha-4A knock-in 45 (Tuba4aKI/KI) mice (from Kneussel lab, ZMNH, Hamburg, Germany), and negative, thus wildtype, littermates (Tuba4a WT/WT) served as controls.
Wild animals	The study did not involve wild animals.
Reporting on sex	Both sexes were included in the study design and information on sex was not collected because synapse elimination is a fundamental developmental process occuring in both sexes equally and at the same scale.
Field-collected samples	The study did not involve field-collected samples.
Ethics oversight	All animal experiments conform to the German federal law. The protocols were examined by the Government of Upper Bavaria and approved by the ethics committee according to §15 German Animal Welfare Act, in agreement with the government of Upper Bavaria. Protocol numbers relevant for this study: ROB-55.2-2532.Vet_02-19-145; ROB-55.2-2532.Vet_02-24-15; ROB-55.2-2532.Vet_02-16-58; ROB-55.2-2532.Vet_02-21-123

Note that full information on the approval of the study protocol must also be provided in the manuscript.

Plants

Seed stocks	n. a.
Novel plant genotypes	n. a.
Authentication	n. a.
